# Is Treating Motor Problems in DCD Just a Matter of Practice and More Practice?

**DOI:** 10.1007/s40474-015-0045-7

**Published:** 2015-04-01

**Authors:** Marina M. Schoemaker, Bouwien C. M. Smits-Engelsman

**Affiliations:** 1 Centre for Human Movement Sciences, University of Groningen, University Medical Centre Groningen, PO Box 30.001, 9700 RB Groningen, The Netherlands; 2 Faculty of Kinesiology and Rehabilitation Sciences, Katholieke Universiteit Leuven, Leuven, Belgium; 3 Department of Health and Rehabilitation Sciences, Faculty of Health Sciences, University of Cape Town, Old Main Building, Groote Schuur Hospital, Cape Town, South Africa

**Keywords:** Developmental coordination disorder, DCD, Motor learning deficit, Intervention, Problem solving, Serious gaming

## Abstract

Developmental coordination disorder (DCD) is often called a motor learning deficit. The question addressed in this paper is whether improvement of motor skills is just a matter of mere practice. Without any kind of intervention, children with DCD do not improve their motor skills generally, whereas they do improve after task-oriented intervention. Merely offering children the opportunity to practice motor skills, for instance by playing active video games, did lead to improved motor performance according to recent research findings, but to a lesser extent than task-oriented intervention. We argue that children with DCD lack the required motor problem-solving skills necessary to further improve their performance. Explicit motor teaching with an emphasis on developing these problem-solving skills is a necessary ingredient of intervention in DCD, leveraging the effectiveness of intervention above that of mere practicing.

## Introduction

According to the *Diagnostic and Statistical Manual of Mental Disorders*, developmental coordination disorder (DCD) refers to those children whose acquisition and execution of motor skills is substantially below their age and opportunities for learning [1]. The coordination problems are expressed in slow and inaccurate performance of motor skills, including activities of daily life, sports, and leisure activities. DCD is sometimes called a motor learning deficit, as these children have difficulties learning to perform all kinds of motor skills in daily life which their typically developing (TD) peers seem to acquire almost effortlessly. TD children learn motor skills either implicitly or explicitly by observing and imitating other children and adults or by trial and error. Important in motor learning is the inherent ability of TD children to monitor their own performance, to detect possible errors, and to identify possible sources of these errors. In addition to the ability to detect and correct errors, the amount of practice with a particular skill, or time-on-task, is an important determinant of improved motor skill in children. For instance, in infants, the amount of experience in locomotion is regarded as the most important determinant of improvements in walking skill [2]. An important question in DCD is whether the motor learning deficit is merely a matter of lack of sufficient practice. Put another way, if we give children with DCD sufficient opportunities to practice motor skills, will their motor problems gradually disappear?

## DCD, a Motor Learning Deficit

Although DCD is characterized by deficits in skill acquisition, remarkably little research has been done in this domain. The few studies that have attempted to study questions relating to deficient or inefficient learning in children with DCD are inconsistent in outcome. It is reported that the children with DCD are delayed in reaching the level of automaticity [3]. An important new part in the description of the DSM 5 criteria is that the child with DCD should have had enough opportunities for learning. Recent theory also acknowledges that contextual factors may play a large role in mediating developmental outcomes and should be taken into consideration early on [4]. To date, no intervention studies are known, which tested if children with DCD only need more time and opportunity to practice in order to reach an appropriate level of performance in motor tasks compared to their TD peers. If that were the case, then specific intervention would not be necessary, but rather providing the child more exercise time with enriched affordances by the home and school environment should suffice. Based on these findings, there is a clear need for rigorous intervention studies using different motor learning paradigms, ranging from simply giving enough opportunity to practice to tailored client-focused interventions. Ideally, these studies should involve not only valid and reliable test outcome measures but also pre- and post-fMRI measurements to look at task-related changes in the brain.

## Decreased Participation in Motor Activities in Children with DCD

The ICF defines participation as the involvement of a person in a life situation [5]. In the case of motor activities, it encompasses involvement in activities of daily living (ADL) or in sports and leisure time motor activities. Participation refers not only to the amount of time a child engages in motor activities but also to the perceived ability to perform well and the motivation to perform an activity [6]. Several studies suggest that children with DCD have an activity deficit, as they participate less in ADL and in both organized and non-organized physical activities [7, 8]. Organized activities include both school-related activities and activities outside school, such as participating in organized sports. Non-organized activities include activities performed during leisure time at home or during recess at school. For instance, observation of the amount of motor activity during recess at school showed that children with DCD were most often onlookers, observing the active play of other children [9]. But also during organized activities such as physical education classes at school, children with DCD engaged more often in off-task behaviors, such as going to the toilet, than on-task behaviors [10]. It is obvious that activity limitations experienced by children with DCD will influence participation in sports or activities in which these fundamental movements are required. But that is not the complete picture. Several other reasons have been put forward for their reduced participation, such as avoidance of failure experiences [11]. Their lower scores in scales measuring perceived physical competence demonstrate that at a certain age these children are well aware of their lack of competence in motor skills [12, 13]. Self-perceptions are an important mediator of physical activity participation [11]. The vicious circle that develops out of avoidance of participation in motor activities is obvious: reduced participation leads to diminished opportunities to practice motor skills, which may result in less opportunity to improve motor skill performance. As a consequence, children with DCD not only become less physically fit but are also found to experience more loneliness as they participate less in social play and sports [14•]. All these factors together may contribute to the development of internalizing symptoms as anxiety and depression in children with DCD [14•, 15].

However, reduced participation is not the only explanation for the motor skill deficits of children with DCD. In a recent, yet unpublished study, parents of children with DCD were asked to fill out the DCDDaily Questionnaire. This questionnaire consists of three scales; parents rate for 23 ADL items how well children are able to perform the activities, whether it took longer to learn the activities, and how often children participate in these activities. According to the parents, children with DCD performed worse in all ADL included in the questionnaire. However, in the majority of those tasks (17 out of 23), they were rated to participate as often as their peers. These ADL included dressing, writing, hopping in squares, and brushing teeth, tasks often mentioned as difficult to perform for a child with DCD. Although we subscribe the necessity of participation or time-on-task for learning a motor skill, the results of the aforementioned study highlight that mere participation may not be enough to improve the level motor skill learning in children with DCD. Other factors may also play a role, such as learning from doing and the problem-solving skills of a child.

## Problem-Solving Abilities of Children with DCD

In order to improve performance, it is necessary that children with DCD develop the right problem-solving skills, such as the ability to identify and correct errors. To this end, it is also important that they possess accurate understanding about the requirements of a motor task [16•]. Several studies have demonstrated that this understanding is often lacking in DCD [17, 18]. Consequently, they often focus on less relevant aspects or incorrect causes of an incorrect motor activity when they try to identify possible causes for their incorrect task performance, for instance by referring to lack of luck as a cause for failure or by stating that the target is too far away when in fact they threw the ball too soft [19]. In general, children with DCD were found to less often plan, monitor, and evaluate their performance [19]. This is in line with a recent study; Hyland and Polatajko reported that children with DCD were able to recognize that their motor performance was not adequate, but they failed to identify the cause of their performance deficit [20]. For that reason, Sangster and Whitebread concluded that intervention should also incorporate the development of problem-solving abilities in children with DCD to enable them to improve their motor skills [16•]. Otherwise, impaired cognitive-motor function may limit their ability to benefit from the interactions with the environment and compromise their psychosocial development. Thus, supportive, enabling environments should create opportunities for motor skill development and promote emotional engagement in physical activity [21].

So we may conclude that treatment of DCD may not just be a matter of offering opportunities to practice motor skills but also of creating an environment in which children can engage in the (physical) activity and learn to detect and correct their motor performance. In the next paragraph, an overview will be given of current treatment methods and their effectiveness.

## Intervention Methods and Their Effectiveness

Over the past 40 years, several treatment methods have been developed, which can be divided roughly into two categories: process-oriented treatment approaches and task-oriented treatment approaches. The main assumption of process-oriented or deficit-oriented approaches is that a deficit in a body structure or sensory process is responsible for the motor skill problems of children with DCD. The aim of treatment is to remediate this deficit, which will result in improved motor task performance. One of the most well-known examples of a process-oriented approach is sensory integration therapy [22]. However, despite its popularity, results of a recent review and meta-analysis of the efficacy of interventions (published between 1995 and 2011) showed that the effect size of process-oriented intervention is weak (0.12) [23••]. The results of this study are in line with those of a comparable meta-analysis summarizing the efficacy of interventions investigated in studies published between 1983 and 1993 [24]. Due to the limited availability of methodologically sound studies, the application of process-oriented approaches (like sensory integration therapy) was not recommended in the recent recommendations of the European Academy of Childhood Disabilities (EACD) on the definition, diagnosis, and intervention of DCD [25••] and not recommended in a policy statement of the American Academy of Pediatrics [26].

Task-oriented approaches focus on teaching those motor tasks that are difficult for a child with DCD, and are designed to improve functional outcomes. For each task, task performance is analyzed in order to identify aspects of the task that are difficult for a child. Recent examples of task-oriented interventions are Neuromotor Task Training (NTT) [27–29] and the Cognitive Orientation to daily Occupational Performance (CO-OP) [30, 31]. NTT is based upon motor learning theory and the ecological approach [23••, 29]. The first step is the identification of those tasks and activities related to participation, which are of greatest concern to the child and his family—these are the target of treatment. By using motor teaching strategies, therapists guide children through the different phases of motor skill learning by gradually increasing task demands. Tasks and environmental constraints that impede successful task performance are identified. Task constraints refer to aspects of the task that restrain a motor activity, such as when a child cannot catch a ball that is thrown with too much force or cannot close a shirt with very small buttons. Environmental constraints refer to aspects of the environment that impede performance, for instance when a child tries to cycle when the wind blows too hard or when people are watching. These task and environmental constraints are manipulated in intervention sessions to provide the opportunity to practice and improve the deficient motor skills. In the early phase of learning, providing simple verbal instruction as to the intended outcome of the skill may be adequate to stimulate practice. Next, the child is provided with augmented feedback (information about their performance from the therapist or other external sources) so that they can improve performance on subsequent practice attempts. Techniques such as guided discovery (ask and not tell) are applied to promote efficient learning, to ensure development of the skill, to influence the child’s motivation to persist with practice, and to encourage the child to reflect on their performance to promote problem-solving skills [29].

CO-OP is a child-centered approach based upon cognitive behavior modification theories, in particular the verbal self-instruction strategy developed by Meichenbaum [32]. It focuses on the acquisition of self-chosen occupational skills. During a CO-OP intervention, a child learns this self-instruction strategy, which enables the child to identify why the performance was not successful and to invent and execute plans to correct their performance (the goal-plan-do-check strategy) [31].

Both NTT and CO-OP proved to be effective task-oriented intervention approaches for children with DCD according to the results of the meta-analysis by Smits-Engelsman et al. [23••]. As a result, both approaches are recommended in the EACD guidelines for DCD [25••]. The common factor in both approaches seems to be the development of meta-cognitive skills during intervention, such as the ability to identify and correct performance problems. In a recent study, Hyland and Polatajko demonstrated that children with DCD learned to improve the ability to self-monitor their performance and to identify and correct errors during a CO-OP intervention [20]. Children not only analyzed their performance more often but were also better able to analyze what was going wrong, for instance when a child fails to write straight and comes up with the solution that a ruler is needed to improve performance. According to the authors, this effect was prompted by providing augmented feedback by the therapist and by using guided discovery, i.e., by asking questions about the performance. These results are in line with those of a study concerning NTT investigating the association of the application of teaching principles with treatment effectiveness. In particular, those teaching principles that enhanced problem-solving abilities proved to be effective, such as asking questions about the children’s task performance and sharing knowledge about how to improve task performance [33]. So, we may conclude that it is not only the increased time to practice motor skills that lies behind the effectiveness of task-oriented approaches but also the development of meta-cognitive problem-solving skills, which the child can draw on to learn other skills.

## A New Development in Intervention

Since the publication of the meta-analysis of the effectiveness of intervention approaches, a new development can be noticed in intervention studies, i.e., the application of serious games. As mentioned before, time-on target is an important ingredient of treatment success. Practicing motor skills during intervention sessions is often not enough to increase motor skill performance. In order to increase treatment effectiveness and to promote transfer of what has been learned to daily life, children are often encouraged by therapists to practice at home. However, children with DCD are often not inclined to engage in physical activities at home. According to a study of Kwan et al. [34], boys with probable DCD reported not enjoying physical activities, and they did not feel that they were able to practice regularly. As a consequence, their motivation to become physically active is low. As mentioned before, both motivation and the perceived ability to perform well are important moderators of participation. The lack of enjoyment and motivation prompted clinicians to consider options that might encourage a more positive attitude towards physical activities. Children will be more inclined to practice the activities if they are enjoyable and if they experience success. Therefore, the need for enjoyment and the need to experience success should be important ingredients of intervention options.

One option is to introduce serious games as part of an intervention. A serious game is the application of an interactive game that can be used for purposes other than mere entertainment, such as rehabilitation. Children in general like to play games, as they are fun and motivating, as often some kind of reward is offered when they perform well. Application of serious games as part of an intervention session or as exercises at home may motivate the children to practice more often and as such may increase the number of hours children with DCD are physically active. Several commercially available games have been developed that can encourage children and adults to be physically active, such as the Nintendo Wii Fit Training, the Kinect, and the EyeToy for PlayStation. Recently, four studies have been conducted to investigate the effectiveness of these games as (part of) an intervention for children with DCD.

In a small pilot study, Hammond et al. investigated the effectiveness of a Wii Fit intervention on motor proficiency and on emotional and behavioral problems of children with DCD [35]. Two groups of children were included: a group of ten children with DCD who played nine Wii Fit games focusing on coordination and balance and a group of eight children who practiced motor skills in groups 1 h per week. Each intervention session lasted 10 min and took place three times a week for a month. Motor abilities were measured with the Bruininks-Oseretsky Test (BOT-2) [36], and emotional and behavioral problems were measured with the Strengths and Difficulties Questionnaire (SDQ) filled out by parents [37]. Significant improvements in motor skills were seen after Wii Fit intervention, not only in skills measuring balance but also fine motor precision and visuo-motor integration, although less pronounced. This demonstrated that improvements obtained during intervention transfer to non-exercised skills. Parents also reported less emotional and behavioral problems after intervention. However, the improvements in motor skills were not maintained after a period of 2.5 months without Wii Fit intervention. Nevertheless, the results of this study are encouraging, as they provide evidence of the immediate effectiveness of a Wii Fit training, its popularity with the children, and its positive effect on their motivation to practice.

In another pilot study, the effectiveness of the PlayStation 2 EyeToy game on motor skills and aspects of physical fitness was explored for 4–6-year-old children with DCD. Nine children referred to physical therapy suspected of DCD were included who played the EyeToy games for 60 min once a week over 10 weeks. Several EyeToy games were played, such as volleyball, bowling, and boot camp, requiring accurate upper-extremity movements that involve motor planning, balance, and eye-hand coordination. Effects of intervention were assessed with the Movement Assessment Battery-2 (MABC2) [38], the Developmental Coordination Disorder Questionnaire (DCDQ) [39], the walking and talking test [40], and the 6-min walk test (6MWT) [41]. Like the earlier study, children’s overall performance on the MABC2 improved after intervention, particularly balance skills. An improvement in daily motor activities was also reported by the parents of the children. Walking speed and walking distance did not increase, however. The lack of effect of intervention on walking endurance may be due to the fact that walking endurance was only practiced in two of the games. An interesting part of this study is that from the fifth intervention session onwards, games were introduced in which children had to play against their parents. Both children and parents enjoyed playing together, and the children exerted more effort when playing with their parents [42]. Although the evidence is scarce, the results of several studies confirm that virtual reality games enhance the motivation to engage in practice. Motivation is particularly enhanced when playing against an opponent. Players were found to perform better in a rehabilitation setting when they played in competition [43]. In general, one of the major advantages of virtual reality games is the opportunity to vary task and environmental constraints: the games offer enriched environments, and children can practice functional movements repeatedly (time-on-task) under different task constraints [43].

Jelsma et al. investigated the effect of the Wii Fit balancing games on the balance skills of 14 children with probable DCD and balance problems [44]. Children practiced the Wii balancing games for 30 min, three times a week for 6 weeks. Eighteen Wii balancing games were available, and children were free to choose the games they wanted to play to increase variability of practice. A second group of 14 children with probable DCD and balance problems was included to serve as a no-treatment control group. Performance was assessed pre-post with the Movement Assessment Battery for Children (MABC2) [38], the Wii Fit ski slalom test (which was not practiced), and three subtests of the BOT-2 (balance, running speed and agility, and bilateral coordination) [36]. After intervention, a positive effect on balance skills was found, as measured with the balance test of the MABC2 and the BOT-2 scales running speed and agility and bilateral coordination. This effect was not found for the no-treatment control group. The effects of the WII intervention were largely task specific, as only those skills improved that were close to the balance tasks trained.

The effects of a Wii Fit training have also been compared to those of NTT [15]. The group receiving NTT consisted of 27 children with DCD and were treated in groups of five to eight children, two times a week for 45–60 min over 9 weeks. A second group of 19 children with DCD underwent Wii Fit training for 6 weeks, three times a week for 30 min. These children practiced various games, such as cycling, skiing, soccer, and skateboarding games as well as five games incorporating arm movements. Effects of treatment were assessed with the MABC2 [38], the Functional Strength Measure (FSM) [45], a hand-held dynometer (HDD) [46], the Muscle Power Sprint Test (MPST) [47], and the 20 Metre Shuttle Run Test (20mSRT) [48]. Although both groups improved on the MABC2, only for the NTT group was this statistically significant. Interestingly, the effects of NTT also transferred to tasks not practiced, such as tasks measuring manual dexterity. Previous studies on the effectiveness of NTT also demonstrated transfer effects to untreated skills, such as handwriting skills [27] and balance [49]. Isometric strength did not improve for either the NTT or Wii Fit group, but anaerobic performance did, for both groups. Taken together, these results suggest that application of serious games such as the Wii Fit might be useful for children with DCD with low cardiorespiratory fitness. The authors conclude that the results of their study support the application of both NTT and Wii training for children with DCD, but the results of NTT were superior.

The results regarding the effectiveness of the application of serious games in intervention in these four studies are promising. An important difference between serious gaming and regular physical or occupational therapy is that children learn to perform motor skills more implicitly during serious gaming, as no formal instruction is part of the training. The increase in performance after playing serious games demonstrates that children with DCD are able to learn implicitly. These findings are in line with those of other clinical groups, such as children with CP who benefit from implicit motor learning [50]. Possible elements that induce the effects of playing serious games are the multiple repetition of tasks, variability of practice, and the provision of augmented feedback about their performance [43]. It is well known from literature about motor learning that these elements enhance the acquisition of motor skills [49]. In addition, children often practice serious games on their own and not with other children. Playing on their own has the advantage that they do not have to be afraid of failing in front of other children. Fear of failure often leads to avoidance of participation in physical activities. When children with DCD can practice on their own, and when the games are fun to play, they will be more motivated to engage in physical activities. On the other hand, playing against children with the same level of disabilities in a therapy setting can enhance their motivation and research findings demonstrate that children perform better in competition [43].

Despite the effectiveness of serious games, the results of Ferguson et al. demonstrate that practicing serious games is effective, but not as effective as a regular task-oriented intervention, such as NTT [15]. As only one study has compared the effectiveness of NTT with those of serious gaming, definite conclusions cannot yet be drawn. However, the results of Ferguson et al. do imply that serious gaming cannot replace regular physical or occupational therapy. An important difference between serious gaming and regular intervention is that learning is more explicit in regular intervention, as therapists provide feedback, but also teach problem-solving skills, such as the goal-plan-do-check strategy in CO-OP, and engage children in guided discovery during both CO-OP and NTT. As mentioned before, children with DCD lack these meta-cognitive problem-solving skills, and teaching these skills seems to be an effective element of regular intervention. The superior effectiveness of regular intervention in comparison to serious gaming may be due to the development of these problem-solving skills during regular intervention. However, serious gaming can be an important complementary intervention, which may enlarge the effectiveness of regular intervention.

## Conclusion

Motor learning necessitates active participation in a variety of motor activities. However, for some children with DCD, participation is affected not only by poor motor learning but also by contextual barriers such as the attitudes and support of others, and self-efficacy beliefs, all of which should be taken into account to prevent an activity disorder. The results of studies evaluating the effectiveness of intervention demonstrate that without any kind of intervention, most children with DCD generally do not improve their motor skills to normal standards. So far, specific task-oriented intervention methods, such as CO-OP and NTT, have proven to be most effective. As well, merely offering the children the opportunity to practice motor skills, for instance by playing serious games, can lead to improved motor performance, but to a lesser extent than task-oriented intervention. What we learn from the success of serious games is the importance of success experiences and practice in a safe environment. Whether serious games can be enlisted to produce sustained effects is an issue for future investigation. Whatever the intervention, explicit motor teaching with an emphasis on developing meta-cognitive problem-solving skills seems to be a necessary ingredient for children with DCD. Furthermore, influencing contextual factors to create circumstances where the children can be active and keep practicing have to be part of the overall approach. Important factors may be to create a support system, which encourages children to stay active over time. For instance, informing parents about the necessity may help them to support their children to practice regularly. To date, only a few studies evaluating the effectiveness of intervention for children with DCD have been conducted, and more research specifically on the best way to deliver the intervention is necessary to come to more definite conclusions. Questions that need to be addressed are how implicit and explicit learning can best be combined in intervention and in which stage of motor learning they may be effective. Also, the long-term effects of serious gaming have yet to be established. So far, we know little about the transfer of the effects of gaming to daily life motor performance. And it is important to investigate whether children continue to practice once the intervention has come to an end.
